# Special Issue: Molecular Ecology, Physiology and Biochemistry of Insects 4.0

**DOI:** 10.3390/ijms26052009

**Published:** 2025-02-25

**Authors:** Klaus H. Hoffmann

**Affiliations:** Animal Ecology I, University of Bayreuth, University Street 30, 95448 Bayreuth, Germany; klaus.hoffmann@uni-bayreuth.de

## 1. Introduction

Insects make up the largest number of species and individuals in the world. However, land use changes, pesticide pollution and advancing climate change are already resulting in a drastic reduction in biodiversity and insect biomass. This threat to insects is very likely to have a direct and indirect impact on humanity’s life. Not only are insects the most important pollinators of crops, 75% of cereals alone depend on insect pollination [1], their function as a direct source of protein for humans is becoming increasingly important. Today, numerous insect species are bred on an industrial scale to make them usable as a source of food for humans and animals [2]. Predatory insects and parasites take over the role of pesticides in plant protection [3]. In medicine, insects are becoming increasingly important as a source of alternatives to antibiotics (e.g., antimicrobial peptides) [4], or as a source of chitin and chitosan to produce nanoparticles, e.g., for wound healing or cancer therapy [5].

Many insect groups show astonishing adaptations to changing environmental conditions. To understand these adaptations and make them usable for the conservation of our insect diversity, in-depth knowledge of biochemistry, physiology and molecular biology is necessary, not only for “model insects”.

The 4th edition of a special issue on this topic since 2019 has now been completed with 15 original papers. A 5th issue with a primarily deadline of February 2025 is in preparation: https://www.mdpi.com/journal/ijms/special_issues/I400XJ46IO (accessed on 20 February 2025).

The fifteen articles from Molecular Ecology, Physiology and Biochemistry 4.0 are two reviews and thirteen research papers. The review by Bartling et al. (2024) (contribution 1) reflects our current knowledge about sublethal effects of pesticides on insect physiology and behavior. The precise effects depend on many different factors, including the insect species, age, sex, caste, physiological condition, as well as the type and concentration of the active ingredients and the exposure route.

The second review (Chen et al., 2023) (contribution 2) reports on Notch signaling, an evolutionarily conserved pathway which functions between adjacent cells to establish their distinct identities. While initially discovered and characterized in the model insect *Drosophila melanogaster*, recent studies across various insect species have revealed the broad involvement of Notch signaling in the formation and patterning of the insect embryo, wing, leg, ovary and several other specific structures.

Four of the research papers deal with mechanisms of adaptation to changes in the biotic and abiotic environment. Several insects adapt to annually fluctuating temperatures via seasonal polyphenism. Weigh and Zhang (2024) (contribution 3) found out that the transcription factor *CcFoxO* mediates the transition from summer form to winter form in the pear psylla, *Cacopsylla chinensis*. Their investigations revealed that *CcFoxO* facilitates the accumulation of triglycerides and glycogen, thereby influencing the transition from summer to winter form by affecting cuticle pigment content, cuticle chitin levels, and cuticle thickness. These insights not only enhance our comprehension of *FoxO* functionality but also offer avenues for environmentally friendly management strategies for the pear pest *C. chinensis*. During diapause, a state of temporarily arrested development, insects require low winter temperatures to suppress their metabolism, conserve energy stores and acquire cold hardiness. Uzelak et al. (2024) investigated the combined effects of thermal stress and the diapause program on the expression of selected genes involved in antioxidant defense and heat shock response in the European corn borer, *Ostrinia nubilalis* (contribution 4). By using qRT-PCR, they showed that response to chronic heat stress is characterized by raised mRNA levels of *grx* and *trx*, two important genes of the antioxidant defense system, as well as of *hsp70* and, somewhat, of *hsp90*, two major heat shock protein genes. Honeybees have an interesting phenomenon, where the larval diets of two different honeybee species are exchanged, resulting in altered phenotypes, named as a honeybee nutritional crossbreeding. The study by Abdelmawla et al. (2024) (contribution 5) investigated the contribution of DNA methylation to the phenotypic alternation of a *Apis mellifera*–*Apis cerana* nutritional crossbreed. The study serves as a model of cross-kingdom epigenetic mechanisms in insect body color induced by environmental factors. Benzyl alcohol (E1519) is an aromatic alcohol used in the pharmaceutical and food industry to protect food products against microorganisms during storage. Aim of the study by Kazek et al. (2024) (contribution 6) was to determine the influence of benzyl alcohol on the defense systems of the wax moth *Galleria mellonella*. Their findings indicate that benzyl alcohol treatment increased the levels of HSP70 and HSP90 and decreased those of HSF1, histamine, and cysteinyl leukotriene. Benzyl alcohol application also increased dismutase level in the hemolymph and lowered those of catalase and 8-OHdG, all important factors in the insect immune system.

Two further articles deal with reactions of the immune system of insects to environmental stimuli. Becchimanzi et al. (2024) (contribution 7) annotated the immune genes of soft scale insects (Hemiptera: Coccidae) for which omics data are publicly available. Their results demonstrate the lack of “classical” immune components from other insect families, peptidoglycan recognition proteins, galectins, thaumatins, and antimicrobial peptides. The results offer a list of promising candidates for developing new control strategies based on the suppression of pests’ immunity. α-Pinene is one of the main defensive components in conifers. *Monochamus alternatus* (Coleoptera: Cerambycidae), a wood borer feeding on Pinaceae plants, relies on its detoxifying enzymes to resist the defensive terpenoid. Xue et al. (2023) (contribution 8) isolated and analyzed the epsilon class *gluthation-S-transferase* gene of *M. alternatus* and showed that it takes part in α-pinene adaptation but does not play a great role in the resistance of *M. alternatus* larvae to α-pinene.

Four articles report on new findings in classical insect biochemistry and physiology. Guo et al. (2023) (contribution 9) compared the ultrastructure (by TEM and fluorescens microscopy) and lipid metabolites (by LC-MS/MS) between virgin and mated bumblebee queens, *Bombus terrestris*. The fat body weight of mated queens significantly increased, and the adipocytes were filled with enlarged lipid droplets. 949 and 748 differential metabolites were identified in the fat body of virgin and mated queens, respectively. Most lipid metabolites were decreased in mated queens, especially some biomembrane components. Malpighian tubules (MTs) are arthropod excretory organs crucial for the osmoregulation, detoxification and excretion of xenobiotics and metabolic wastes, which include tryptophan degradation products along the kynurenine (KYN) pathway. Early investigations in *Drosophila* larval fat bodies revealed an intracellular autofluorescence (AF) that depended on tryptophan administration. Subsequent observations documented AF changes in the MTs of *Drosophila* eye-color mutants genetically affecting the conversion of tryptophan to KYN or 3-hydroxy kynurenine. Croce et al. (2024) (contribution 10) characterized the AF properties of the MTs in different stages of the Asian tiger mosquito, *Aedes albopictus*. The findings suggest AF can serve as a promising marker for investigating the functional status of MTs in response to metabolic alterations, contributing to the use of MTs as a potential research model in biomedicine. The pine wood nematode (PWN) uses several *Monochamus* (Cerambycidae) species as vehicles, through a temporary hitchhiking process known as phoresy, enabling it to access new host plant resources. Li et al. (2024) (contribution 11) showed that phoretic interactions between vector beetles and PWN vary throughout the vector’s lifespan, particularly before and after entry of the nematode into the trachea of the long-horn beetle. The study highlights the fitness costs of immunity and metabolism on the vector beetle, indicating the adaptation mechanisms and evolutionary trade-offs to the nematode. Xu et al. (2024) characterized an odorant receptor, PstrOR17, on the antenna of one of the most destructive pests in Brassicaceae plants worldwide, the striped flea beetle *Phyllotreta striolata* (contribution 12). They identified two odorants, (S)-cis-verbenol and (−)-verbenone, which displayed significant attraction for *P. striolata*. RNA interference (RNAi) was used to confirm that PstrOR17 is essential for the detection of (−)-verbenone and (S)-cis-verbenol to elicit an attraction effect. The results lay a foundation for the development of new and effective nonchemical insecticide strategies based on (S)-cis-verbenol and (−)-verbenone.

RNAi, as a gene silencing mechanism is widely conserved across Eukaryota and used to study gene functions, but also for managing insect pests. Prates et al. (2024) (contribution 13) evaluated a range of RNAi-inducing molecules (siRNAs, shRNAs, and dsRNAs) and administration methods (oral delivery, immersion, and microinjection) in three different laboratories. They also tested various mosquito strains and utilized microorganisms for RNA delivery. Their results reveal a pronounced inconsistency in RNAi efficacy, characterized by minimal effects on larval survival and gene expression levels in most instances despite strong published effects for the tested targets. The findings emphasize the intricacies of RNAi application in mosquitoes, which present a substantial barrier to their utilization in genetic control strategies.

Park et al. (2024) (contribution 14) established a relationship between disease-causing microorganisms and sepsis in the silkworm *Bombyx mori*. After producing a 16S rRNA amplicon library for samples showing sepsis, they obtained information on the microbial community present in silkworms using next-generation sequencing. Compared to that in healthy silkworms, in animals with sepsis, the abundance of the *Firmicutes* phylum was significantly reduced, while that of *Proteobacteria* was increased. *Serratia* sp. was dominant in silkworms with sepsis.

In the last paper, Mora et al. (2024) (contribution 15) utilized Illumina next-generation sequencing data from the two-spotted ladybug, *Adalia bipunctata*, to investigate its satellitome. Specifically, they employed the CHRISMAPP pipeline to map the satDNAs of *A. bipunctata* onto the genome of *Adalia decempunctata*, which has also been sequenced and assembled at the chromosome level. The interspecific comparative study provides a significant advance in the understanding of the repeat genome organization and evolution in beetles.

We hope that research on the physiology, biochemistry and molecular ecology of insects will contribute to preserving the diversity of insects on our planet but also contribute to developing insects as a source of food and feed for animals and humans, as a source of novel pharmaceuticals or to find raw materials for human and veterinary therapeutics.
